# Symptom Terminology Normalization in Traditional Chinese Medicine: Development and Evaluation of a 2-Stage Deep Learning Framework Based on Fine-Grained Semantic Classification

**DOI:** 10.2196/85825

**Published:** 2026-09-25

**Authors:** Junyu Yao, Xingyue Gou, Wei Lai, Yuzhu Gao, Siqi Wang, Chuangan Zhou, Hui Ye, Jing Tian, Jun Yi, Dong Cao

**Affiliations:** 1 School of Medical Information Engineering, Guangzhou University of Chinese Medicine, 232 Outer Ring East Road, Guangzhou University City, Panyu District, Guangzhou, Guangdong, 510006, China, 86 13246852860; 2 Yunkang School of Medicine and Health, Guangzhou Nanfang College, Guangzhou, Guangdong, China; 3 School of Foreign Studies, Guangzhou University of Chinese Medicine, Guangzhou, Guangdong, China; 4 School of Medical Information Engineering, Guangdong Pharmaceutical University, Guangzhou, Guangdong, China

**Keywords:** symptom terminology, traditional Chinese medicine, natural language processing, named entity recognition, named entity normalization, pretrained language model

## Abstract

**Background:**

Due to the heterogeneity of symptom terminology and the lack of industry standards, the same symptom is often described using multiple expressions. Current normalization approaches struggle to comprehensively retrieve standard terms when a raw term maps to multiple symptoms.

**Objective:**

This study aimed to address the lack of industry standards for traditional Chinese medicine (TCM) symptom terminology. This study proposed the split-then-concatenate normalization framework (STC-NF), a novel approach based on fine-grained semantic classification and a 2-stage deep learning architecture that uses electronic medical records (EMRs) as the data source.

**Methods:**

This study proposed a 2-stage deep learning framework, “split-then-concatenate.” In the splitting stage, TCM symptom entities were categorized into 12 fine-grained semantic labels, and 3 named entity recognition (NER) models were trained to extract TCM symptom terminology from EMRs. In the concatenation stage, standard terms with the same concept as raw terms were identified using a Bidirectional Encoder Representations from Transformers (BERT)–based binary classification model. The standard terms with specific semantic labels were concatenated and reordered according to predefined rules to output structured text, thereby normalizing TCM symptom terminology.

**Results:**

The proposed STC-NF model achieved an accuracy of 91.4% (180/197) and an *F*_1_-score of 360 out of 389 (92.5%) on the single-implication test set. For multi-implication terms, STC-NF achieved an accuracy of 84.3% (311/369) and an *F*_1_-score of 1958 out of 2316 (84.5%), outperforming sequence generation in accuracy by 33.1 percentage points. On the mixed test set containing both single- and multi-implication terms, STC-NF achieved an accuracy of 88.1% (990/1124) and an *F*_1_-score of 3862 out of 4385 (88.1%), exceeding the best-performing baseline model, multi-task candidate generator (MTCG), by 16.7 and 22.2 percentage points in accuracy and *F*_1_-score, respectively.

**Conclusions:**

In this study, we verified that the fine-grained semantic classification and the 2-stage “split-then-concatenate” framework effectively improved performance of named entity recognition and entity alignment, providing an improved approach to normalizing TCM symptom terminology.

## Introduction

### Background and Rationale

Symptoms are the core basis of traditional Chinese medicine (TCM) identification and treatment. They are also one of the main input features for AI to realize assisted diagnosis and treatment [1], which are of great value to medical research [2]. Due to the heterogeneity of symptom terminology, the same symptom is often described in multiple expressions. For example, “失眠 (insomnia),” “不寐 (sleeplessness),” and “入睡困难 (difficulty initiating sleep)” are all described as the same clinical phenomenon in TCM electronic medical records (EMRs). In recent years, it has been widely acknowledged that normalizing symptom terminology is crucial for improving the accuracy of TCM syndrome differentiation and promoting the modernization of TCM [3]. However, neither industry consensus nor a national standard for TCM symptom terminology currently exists [4]. This gap has significantly hindered the sharing of medical data, knowledge mining, and the development of intelligent applications [5]. Efficiently and accurately aligning nonnormalized symptom raw terms with standard terms in the knowledge base has become a key issue to address.

### Related Work

A number of recent studies have specifically targeted the normalization of TCM symptom terminology. Jia et al [6] proposed a symptom terminology normalization approach with hierarchical semantics (symNormHS) to address issues related to synonymous expressions and homographs through a 2-step process. The approach first extracted hierarchical semantic information from symptom terms using a multilabel text classifier to filter the candidate set. Then, it used a hybrid multigranularity text matching model, combined with an attention mechanism, to compute the similarity between raw terms and standard terms. Zhan et al [7] used an autostacker classifier model based on the automated machine learning (AutoML) framework to predict categories of TCM symptom terms, and computed semantic similarity between raw terms and standard terms using a directional skip-gram (DSG) model. Tang et al [8] retrieved candidate standard terms from Systematized Nomenclature of Medicine Clinical Terms (SNOMED CT) using a hybrid retrieval strategy that combined dice coefficients and term frequency-inverse document frequency (TF-IDF). The retrieved candidates were then reranked using enhanced representation through knowledge integration (ERNIE), specifically the ERNIE-Health model (Baidu Inc), which served as the semantic similarity scoring module for selecting the most semantically compatible standard terms. Zhou et al [9] unified the synonymous raw terms into standard terms by constructing a classification model for synonymous term conversion with the thinking of candidate terms (STC-TC), and misclassification detection was performed using the output comparison of multiple heterogeneous models (OCMH). Hu et al [10] proposed an approach to normalize TCM symptom terminology using Bidirectional Encoder Representations from Transformers (BERT)–bidirectional long short-term memory (BiLSTM)–conditional random field (CRF) and a multilabel classification strategy. By constructing an ontology framework for TCM symptoms and integrating knowledge graphs, this approach combined the correlated feature fusion module (CFFM) and the hierarchical labeling tree (HLT) to optimize the recognition and normalization capability of entities. The normalization approaches in [6-10] are all based on deep learning. Their innovations and limitations are summarized in Table 1.

**Table 1.** Innovations and limitations of different approaches.

Author	Year	Approach	Innovations	Limitations
Jia et al [6]	2021	symNormHS^a^	Enhanced semantic matching by fusing unigram- and bigram-level local features through the attention mechanism.	Low recall of multi-implication terms^b^ (eg, “面红目赤 [flushed face with bloodshot eyes]” only matched “目赤 [bloodshot eyes]”), which required improving the normalization logic by formulating additional heuristic rules.
Zhan et al [7]	2021	DSG^c^	Constructed a classification model to handle symptom terms using the AutoML^d^ framework and used the DSG model to capture the semantic relationships between raw terms and standard terms.	The DSG model had a limited ability to recognize multi-implication terms (eg, “头胀痛伴耳鸣如蝉 [distending headache with tinnitus like cicada chirping]”), which might lead to normalization failure due to excessively long text and semantic nesting.
Tang et al [8]	2022	ERNIE^e^-Health (Baidu)	Proposed a hybrid recall strategy (dice coefficient+TF-IDF^f^) to improve the recall of standard terms.	Unable to accurately deal with multi-implication terms (original description: 2 different concepts in one word), it was suggested to introduce external information, such as “context and symptomatic site,” to address this issue.
Zhou et al [9]	2023	STC-TC^g^	Used the OCMH^h^ to identify normalization errors.	Multi-implication terms (eg, “痰量多色白 [increased white-colored sputum]”) had lower normalization accuracy due to their extremely low frequency of occurrence and the lack of sufficient training samples for the model to learn entity recognition laws.
Hu et al [10]	2024	Ontology-enhanced multilabel model	Introduced the CFFM^i^ module to fuse entity features and the hierarchical labeling tree to solve the label imbalance problem, thereby enhancing normalization capability for rare symptoms.	The multilabel classification approach was unable to handle multi-implication terms composed of multiple symptom entities (eg, “腰膝酸软 [soreness and tenderness in the lower back and knees]”), as it only matched “腰酸软 (lower back soreness and tenderness)” and could not retrieve “膝酸软 (knee soreness and tenderness).”

a symNormHS: symptom terminology normalization approach with hierarchical semantics.

b In this study, we define a raw term that maps to exactly one standard term as a “single-implication term,” and one that maps to multiple standard terms as a “multi-implication term.”

c DSG: directional skip-gram.

d AutoML: automated machine learning.

e ERNIE: enhanced representation through knowledge integration.

f TF-IDF: term frequency-inverse document frequency.

g STC-TC: synonymous term conversion with thinking of candidate terms.

h OCMH: output comparison of multiple heterogeneous models.

i CFFM: correlated feature fusion module.

Beyond these TCM-specific systems, research on medical terminology normalization has more broadly progressed through 3 methodological stages, including rule-based and dictionary-matching approaches, machine learning approaches, and deep learning approaches. Rule-based and dictionary-matching approaches [11-16] require experts to manually construct specific regulations based on data features and then use specialized domain dictionaries to complete tasks such as named entity recognition (NER) and named entity normalization (NEN). Although such approaches achieve high accuracy when the lexicon is comprehensive, their performance is contingent on labor-intensive, manually crafted rules. Furthermore, reusing identical rule sets in new domains markedly reduces effectiveness, rendering the approach costly, laborious, and poorly portable. Machine learning approaches [17-22] can automatically extract features from medical corpora and predict entity classification labels through statistical computation, thereby eliminating the need to construct rules and effectively reducing manual effort. However, this approach computes the similarity of concepts by comparing the literal differences between 2 entities, which does not fully use the semantic information in the context, resulting in obvious limitations in its generalizability. Deep learning approaches [6-10,23-32] now dominate medical terminology normalization because they can embed phrases in dense vector spaces and capture latent semantic relations beyond surface forms. Recent studies have further shown that medical NER and normalization are affected by limited annotated data, entity-boundary ambiguity, heterogeneous preprocessing procedures, and insufficient use of contextual information. For Chinese clinical NER, Tang et al [33] proposed a segmentation synonym sentence synthesis mechanism to expand training data and improve model generalization. Li et al [34] explored a few-shot medical NER framework that combines retrieval-based example selection with a structured reasoning process, highlighting the importance of informative examples and task-specific guidance under limited annotation conditions. From the perspective of biomedical entity linking, Garda et al [35] emphasized that inconsistent preprocessing, knowledge-base selection, and evaluation protocols can limit reproducibility and comparability across normalization systems. Luo et al [36] further demonstrated that contextual information around entity mentions is important for biomedical named entity normalization, especially when mentions are semantically ambiguous. These studies collectively indicate that accurate clinical text standardization requires not only semantic matching but also reliable entity-boundary recognition, task-specific preprocessing, and context-aware alignment. To address these requirements from different methodological perspectives, existing deep learning approaches can be broadly categorized into 3 types based on their entity alignment mechanisms, including multiclassification, sequence generation, and binary classification.

The multiclassification paradigm casts normalization as a sentence-level classification task. Representatively, Huang et al [30] proposed a pretrained language model framework for *International Classification of Diseases* coding (PLM-*ICD*), which integrates domain-specific pretrained language models with the attention mechanism and thereby exploits contextual semantics to align uncommon raw terms (eg, “耳朵有蝉叫声 [ears buzzing]”) with standard terms of low lexical overlap (eg, “耳鸣 [tinnitus]”). Compared with statistical models such as TF-IDF, Med, and BM25, which rely on character-level similarity, this paradigm attains markedly higher accuracy on single-implication terms; however, because its classification head emits only a single prediction for each input sentence, it cannot recover the multiple standard terms that a multi-implication expression requires. To overcome this single-output limitation, the sequence generation paradigm reformulates normalization as a token-by-token generation process. Along this line, Yan et al [31] generated standard terms sequentially and were thus able to align simply structured multi-implication terms that contain no overlapping, discontinuous, or nested entities (eg, “头昏嘴斜 [dizziness and mouth skewed]”) with several standard terms (eg, “头晕 [dizziness]” and “口歪 [mouth skewed]”), thereby partially improving the accuracy of multi-implication normalization. Decomposing a raw term character by character, however, can fracture the structural integrity of an entity and degrade performance on single-implication terms. The binary classification paradigm instead preserves each candidate term intact and judges semantic equivalence in a pairwise manner. For example, Liang et al [32] proposed the multi-task candidate generator (MTCG) model, which first retrieves the standard terms semantically closest to a raw term by computing the Euclidean distance between their vector representations, and then formats each raw term together with its retrieved candidates as sentence-pair inputs to a pretrained BERT model for binary classification; an output of 1 indicated semantic equivalence between the raw term and a candidate standard term, and the normalization of a multi-implication term is completed by aggregating all candidates judged to be equivalent.

### Challenges in TCM Symptom Terminology Normalization

While the 3 paradigms reviewed above each advance entity alignment, none was designed to fully decompose the structurally complex symptom expressions that are common in TCM EMRs. As shown in Table 1, deep learning approaches can retrieve standard terms with low character-level similarity to raw terms—for instance, mapping “脑袋疼 (nǎo dai téng)” to “头痛 (tóu tòng),” both meaning “headache”—by capturing linguistic features and modeling contextual dependencies. However, for multi-implication terms composed of multiple symptom entities, current normalization approaches still perform with low accuracy. To better understand this limitation, after manually annotating 500 TCM EMRs, we found that multi-implication terms related to the TCM symptom terminology could be categorized into the following three types.

1. Overlapping entities: two symptom terms share the same suffix. For instance, the phrase “饮食睡眠尚可 (eating and sleeping okay)” is a combination of the symptom terms “饮食尚可 (eating okay)” and “睡眠尚可 (sleeping okay).”
2. Discontinuous entities: common TCM symptom terminology, such as “头痛 (headache),” “手麻 (hand numbness),” and “耳鸣 (tinnitus),” typically consists of an anatomy entity and an abnormality entity. However, sometimes the abnormality entity does not follow the anatomy entity. For instance, in the phrase “头有点痛 (a mild headache),” the adjective “有点 (mild)” (indicating symptom severity) separates the abnormality entity “痛 (pain)” from the anatomy entity “头 (head).”
3. Nested entities: two symptom terms share the same prefix. For instance, the phrase “尿频量多 (polyuria with increased urinary frequency)” results from combining symptom terms “尿频 (frequent urination)” and “尿量多 (copious urination).”

These 3 complex structural types are the main factors responsible for the low accuracy of current normalization approaches in handling multi-implication terms. To improve normalization performance for such terms, this study proposes the split-then-concatenate normalization framework (STC-NF), an approach based on fine-grained semantic classification that uses TCM EMRs as the data source.

### Study Objective and Contributions

Guided by the objective of accurately normalizing such multi-implication terms, this study makes the following two principal contributions:

1. Fine-grained semantic classification rules are proposed to categorize entities related to TCM symptoms into 12 different types. Validation on 500 TCM EMRs demonstrates that labeling data with fine-grained semantic classification enables the NER model to achieve excellent performance. This approach could effectively address the difficulty in segmenting TCM symptom terminology caused by fuzzy entity boundaries.
2. Concatenation and ordering rules for standard terms are constructed to retrieve standard terms that semantically match the input raw term from the knowledge base more comprehensively when the latter maps to multiple standard terms. This approach shows excellent performance in handling multi-implication terms and could be applied to other medically relevant natural language processing (NLP) tasks, such as normalizing diagnoses or surgical procedures.

## Methods

### Overview of the Study

**Figure 1.**
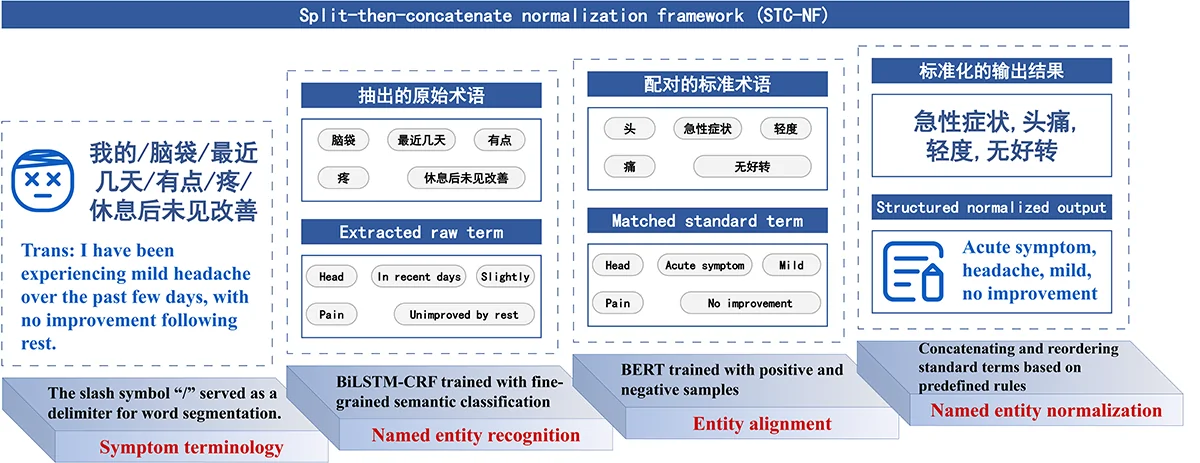
Technical roadmap for normalization of traditional Chinese medicine (TCM) symptom terminology. BERT: Bidirectional Encoder Representations from Transformers; BiLSTM: bidirectional long short-term memory; CRF: conditional random field.

To accurately extract all symptom terms from EMRs and convert them into structured text, we proposed the STC-NF. This 2-stage process was designed after reviewing 3 normalization approaches, including multiclassification, sequence generation, and binary classification. In the first stage, we segmented sentences and extracted all symptom-related raw terms using fine-grained entity classification rules and a BiLSTM-CRF model. In the second stage, our study used a binary judgment model based on BERT to identify the conceptually equivalent standard term from a set of standard terms that shared the same semantic classification label as the raw term. Finally, we concatenated and reordered all standard terms according to predefined rules to generate the structured text, thereby completing the normalization of the entire sentence. The technical workflow of this study is shown in Figure 1.

### Introduction to Dataset Sources and Fine-Grained Semantic Classification

The primary source of the dataset in this study was the collection of 500 EMRs from the endocrine department of a hospital in Guangzhou, China. Before annotation, personally identifiable information was removed, and the free-text symptom descriptions were reviewed at the sentence level. Because this study used manually annotated free-text symptom mentions rather than structured laboratory or demographic variables as model inputs, conventional missing-value imputation was not performed. Sentences within the EMRs that lacked analyzable symptom descriptions were excluded from training instance construction; this sentence-level filtering did not affect the EMR-level dataset partitioning described below. The main features used in the NER stage were entity boundaries and fine-grained semantic labels, while the features used in the NEN stage were raw-term and candidate-standard-term pairs constrained by semantic label type. This design is consistent with recent clinical NER and biomedical entity-linking studies emphasizing task-specific annotation rules, limited-resource data construction, and standardized preprocessing for reproducible normalization experiments [33-35]. We categorized the symptom-related entities into 12 different types, and the specific classification rules and entity examples were as follows:

1. Independent symptom (label: Symp_S): a highly condensed name for a disease or an expression describing a preference, usually consisting of 2 Chinese characters. Examples: “咳嗽 (cough),” “呕吐 (vomiting),” “发热 (fever),” and “喜热饮 (preference for hot drinks).”
2. Negative occurrence (label: Neg): a character or word that describes a negative meaning. Examples: “无 (none)” and “未见 (not seen).”
3. Scope space (label: Pos_SCP): describes the scope in which the state occurs. Examples: “左 (left),” “右 (right),” “双侧 (bilateral),” and “小 (the character ‘小 [xiǎo]’ in the TCM symptom terminology ‘小便 [xiǎo biàn]’ implies ‘a relation to urination or the urinary system’).”
4. Primary space (label: Pos_Pri): describes the primary space in which the state occurs. Examples: “头 (head),” “神志 (mental),” “睡眠 (sleep),” and “便 (poop).”
5. Secondary space (label: Pos_Sub): after splitting the compound entity, describes a more specific level of the state. Examples: “量 (volume)” and “次数 (frequency)” in “小便量次数增多 (increased urinary frequency with elevated voided volume).”
6. Time of occurrence (label: Time): describes the time of occurrence of all abnormal symptoms. Examples: “三个月前 (3 months ago)” and “昨日 (yesterday).”
7. Occurrence condition (label: Cond): describes the specific time or condition under which an independent symptom occurs. Examples: “行走时 (when walking),” “平躺时 (when lying down),” “晨起时 (when waking up in the morning),” and “上楼后 (after going upstairs).”
8. Frequency of occurrence (label: Freq): describes how frequently the symptom occurs. Examples: “一日三次 (3 times a day),” “反复 (recurrent),” and “偶尔 (occasional).”
9. Qualitative severity (label: Sev_Qual): describes the qualitative seriousness of the symptom. Examples: “轻度 (mild),” “中度 (moderate),” and “重度 (severe).”
10. Quantitative severity (label: Sev_Quant): describes the quantitative severity of the symptom quantitatively. Examples: “约50斤 (about 25 kg)” in “体重下降约50斤 (significant body weight reduction of approximately 25 kg).”
11. Occurrence state (label: State): describes the abnormal manifestation of the symptom. Examples: “痛 (pain),” “刺痛 (stabbing pain),” “闷 (stuffiness),” “胀 (bloating).”
12. Occurrence trend (label: Trend): describes the trend in symptom change. Examples: “加重 (aggravation),” “减轻 (remission),” “缓解 (alleviation).”

All 500 EMRs were annotated according to these 12 fine-grained categories before model training and evaluation. The dataset was divided at a ratio of 8:2. A total of 400 EMRs, accounting for 30,490 out of 38,486 entities (79.2%), were used as the training set, and 100 EMRs, accounting for 7996 out of 38,486 entities (20.8%), were used as the test set.

### Overview of the Entity Alignment Task

Entity alignment for symptom terminology aims to map each fine-grained raw symptom term extracted from medical texts to a semantically equivalent standard term in a standard knowledge base. Given a set containing n raw terms:


(1) $$M=\left\{m_{1},m_{2},\cdots ,m_{n}\right\}$$

and a standard knowledge base (containing *g* standard terms):


(2) $$E=\left\{e_{1},e_{2},\cdots ,e_{g}\right\}$$

The task is to map each fine-grained raw term m_i_ to a semantically equivalent standard term e_j_ in the standard knowledge base, with the candidate standard terms restricted to those sharing the same fine-grained semantic label as the raw term.

The core methodology involves training a deep semantic matching model, leveraging BERT’s binary classification capability, to perform the normalization shown in Equation 3:


(3) $$norm\;(m_{i})=e_{j}$$

For a multi-implication symptom term, this mapping is applied to each of its extracted fine-grained raw terms, and the matched standard terms are subsequently concatenated and reordered according to predefined rules.

**Figure 2.**
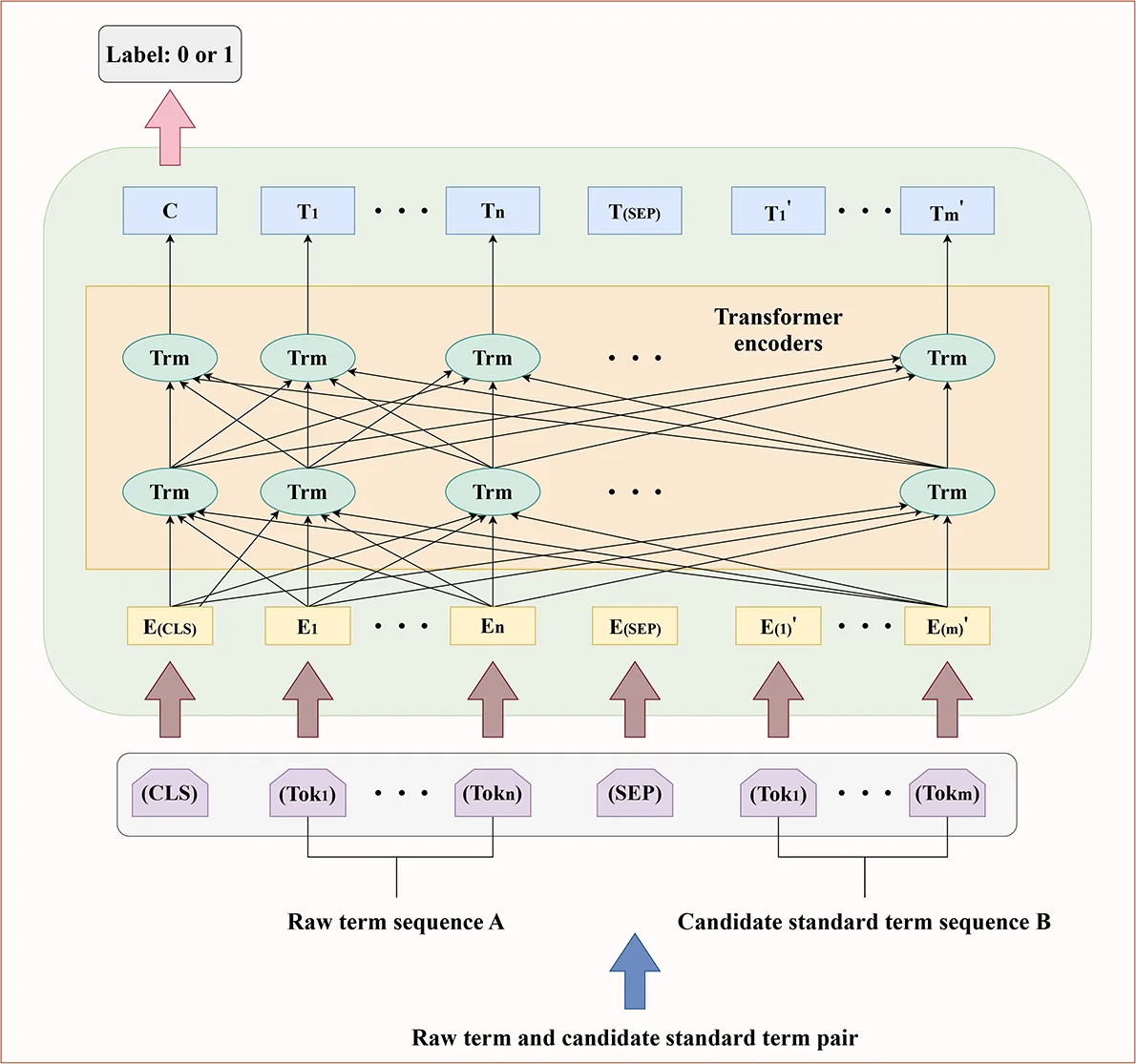
Architecture of a Bidirectional Encoder Representations from Transformers (BERT)–based model for entity alignment. CLS: classification token; SEP: separator token; Tok: token; Trm: transformer module.

As illustrated in Figure 2, our proposed model processes a pair consisting of a raw term and a candidate standard term. The (CLS) token is placed at the beginning of the input sequence for the final text classification task, while the (SEP) token is used to separate the 2 terms. The model outputs a binary classification result: a label of “1” indicates a conceptual match, signifying that the candidate standard term is the correct alignment for the given raw term. Conversely, a label of “0” denotes a mismatch, meaning the candidate term is not the correct normalization. In such cases, the model iteratively evaluates the raw term against other candidate standard terms until a positive match (a result of “1”) is identified.

### Introduction to the Methodology for Constructing the Standard Knowledge Base

Most of the standard terms we collected originate from the most widely used medical classifications and databases worldwide. For example, the terms “失眠 (insomnia),” “不寐 (sleeplessness),” and “辗转反侧 (nocturnal restlessness)” all convey the same concept and describe the symptom of “睡眠欠佳 (sleep disturbance).” However, only “失眠 (insomnia)” has a corresponding disease code in the *International Statistical Classification of Diseases, 10th Revision* (*ICD-10*) codes, so we set “失眠 (insomnia)” as the standard term. Under our fine-grained semantic classification rules, “失眠 (insomnia)” is categorized as the Symp_S entity. By aggregating such standard terms with the Symp_S label, we constructed the Symp_S standard knowledge base. In this study, symptom-related entities are categorized into 12 distinct types, necessitating the construction of 12 separate standard knowledge bases. Due to the limitations of the article’s length, only the sources for the Symp_S and Pos_Pri standard terms are introduced.

We first describe the Symp_S standard knowledge base. In the field of medical information, the *ICD-10*, developed by the World Health Organization (WHO), has been widely used as a diagnostic coding and disease classification system in health records, information exchange, research, quality measurement, and payment applications [37]. We collected 136 Symp_S standard terms using *ICD-10* codes as the reference.

We then describe the Pos_Pri standard knowledge base. SNOMED CT is a comprehensive international clinical terminology system that contains more than 350,000 concepts and is organized into 19 top-level hierarchies, including body structure and clinical findings [38,39]. Because Pos_Pri entities are used to describe the primary site of a symptom, we constructed the Pos_Pri standard knowledge base by referring to the body structure hierarchy of SNOMED CT. The Pos_Pri standard knowledge base currently contains 106 commonly used anatomical terms.

Finally, we describe how the standard knowledge bases for the infrequent entity types were constructed. Entity types such as Time, Cond, Freq, and Trend are used less frequently in medical records. After searching for *ICD-10*, SNOMED CT, and the relevant literature on entity normalization, we were unable to find a dataset of these uncommon types of entities. To construct standard knowledge bases for these infrequent entities, we counted the number of occurrences of each extraction result after recognizing entities in 500 EMRs. Entities that occurred more than 10 times were designated as standard terms, after which domain experts manually removed duplicates with identical semantics.

### Introduction to the Method of Constructing Positive and Negative Samples

We begin with the construction of positive samples. First, an NER model was used to extract all symptom entities contained in the sentence. Then, based on the label types of the symptom entities, experts manually identified the standard term with the same concept from the corresponding standard knowledge base. For instance, in the sentence “我的脑袋最近几天针扎样痛 (My head has had lancinating pain for several days),” the raw term “脑袋 (head)” was identified as a Pos_Pri entity. Therefore, to construct a positive sample about the entity “脑袋 (head),” we needed to find a synonym for “脑袋 (head)” in the Pos_Pri standard knowledge base. A detailed list of raw terms and their corresponding standard terms used for positive sample construction is available in Multimedia Appendix 1.

We then turn to the construction of negative samples. Negative samples were generated in batches by randomly combining the raw terms with unmatched standard terms of the same type. Take the sentence “我的胸口有刀割样痛 (I have a lancinating pain in my chest)” as an example. According to the result of named entity recognition, “刀割样痛 (lancinating pain)” was categorized as a State entity. The matched standard term in the State standard knowledge base was “绞痛 (colic),” while “酸痛 (soreness)” and “刺痛 (stabbing pain)” were unmatched standard terms in the same standard knowledge base. By combining “刀割样痛 (lancinating pain)” with “酸痛 (soreness)” and “刺痛 (stabbing pain),” respectively, negative samples were constructed for training the binary classification model.

The manual pairing required for positive samples (described above) contrasts with the batch generation possible for negative samples, which can lead to an imbalanced dataset. To address this potential imbalance, we used 5 data augmentation methods specifically to expand the positive sample set. The operational details and examples of these methods are provided in Multimedia Appendix 2.

We now present concrete examples of the constructed samples. We constructed a total of 3350 positive and negative samples. The ratio of positive to negative samples was 1:1. Examples are shown in Table 2, where label 1 represents that the raw term has the same concept as the standard term. Label 0 represents that the concepts of the 2 terms are different.

**Table 2.** Examples of positive and negative samples for the entity alignment model.

Raw term	Standard term	Label
Nǎo dai (脑袋)	Head	1
Pain tolerable, not affecting daily life or sleep quality, able to work	Mild [40]	1
Severe pain, unbearable, requiring analgesics, sleep quality impaired, but still able to continue working	Moderate [40]	1
Severe pain, unbearable, requiring analgesics, sleep severely disturbed, unable to work, requiring hospitalization for systematic treatment	Severe [40]	1
24-hour urine output ≥3000 mL [41]	Polyuria	1
Hourly urine output ≤17 mL [41]	Oliguria	1
24-hour urine output ≤100 mL [42]	Anuria	1
Bowel movements fewer than 3 times per week [43]	Constipation	1
Bowel movements more than 3 times per day [44]	Diarrhea	1
Within the past few days	Acute symptoms	1
Occurring after climbing stairs	Exertion-induced	1
Lancinating pain	Colicky pain	1
Lancinating pain	Numbness	0
Lancinating pain	Aching pain	0

### Methods of Normalizing Symptom Terminology

**Figure 3.**
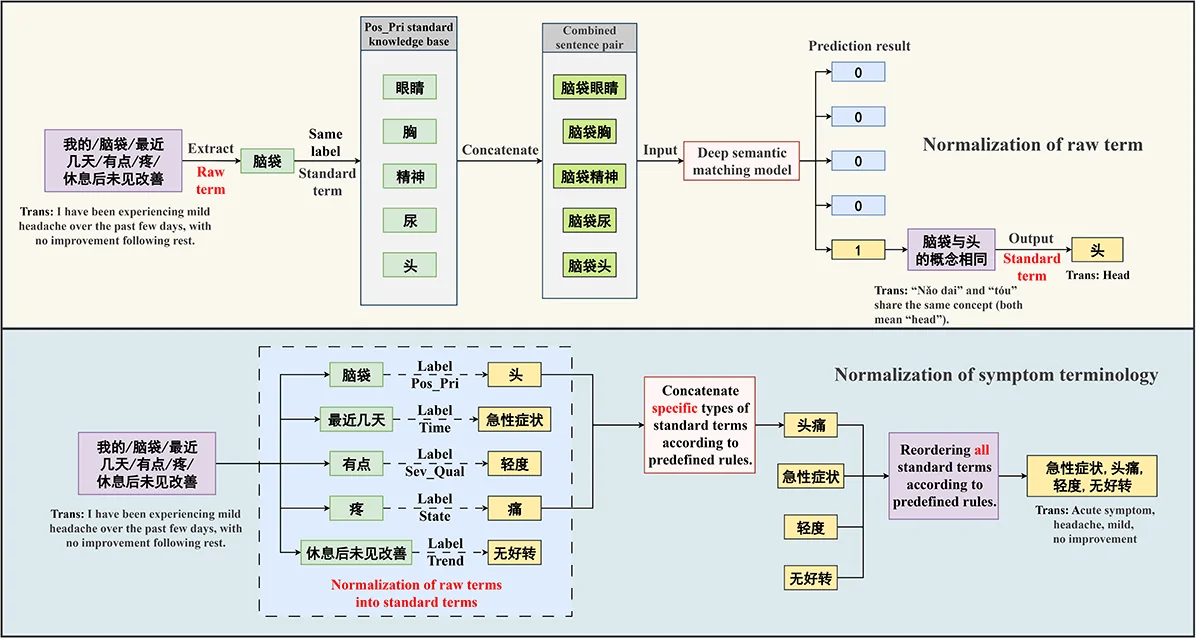
Normalization methods for raw terms and symptom terminology.

After inputting a paragraph of symptom terminology, the NER model was first used to extract raw terms. Each extracted raw term was paired with every standard term sharing the same fine-grained semantic label. Restricting candidate standard terms to the same semantic label reduced unnecessary comparisons across clinically incompatible entity types and made the alignment process more interpretable. This strategy is also consistent with the general formulation of biomedical entity linking, in which an entity mention is mapped to a standard concept or term in a predefined knowledge base [35]. Then, the binary classification model was used to determine whether the sentence pairs, obtained by concatenating the raw term and the standard term, represented a semantic match (label 1) or a nonmatch (label 0). Consistent with evidence that surface-form overlap is insufficient for resolving semantically ambiguous biomedical mentions [36], the alignment model was designed to judge semantic equivalence between raw terms and candidate standard terms rather than relying only on surface-level string similarity. A prediction of 1 signified semantic equivalence, prompting the system to output the corresponding standard term. If all predictions were 0, the raw term (eg, a Sev_Quant entity such as “about 5 kg”) was retained as the standard term because no matching result was found in the knowledge base. After normalizing all raw terms, standard terms were concatenated and reordered according to predefined rules to generate the final standardized symptom description. The normalization methods of raw terms and symptom terminology are shown in Figure 3.

### Concatenation Rules for Standard Terms

To extract and reconstruct comprehensive symptoms from complex expressions with overlapping, discontinuous, and nested entities, after the deep semantic matching model normalized each extracted raw term to a standard term, we concatenated 6 categories of standard terms, including Neg, Symp_S, Pos_SCP, Pos_Pri, Pos_Sub, and State. This rule-guided reconstruction step was necessary because multi-implication TCM symptom expressions may contain more than one clinically meaningful concept in a single raw phrase. Unlike conventional entity-linking settings that often assume a fixed mention span and map it to one knowledge-base concept [35], our task required both fine-grained decomposition of symptom components and structured recombination of multiple normalized components. After studying the raw medical records, we summarized the concatenation rules for standard terms into the following 5 types. The slash symbol “/” served as a delimiter between words.

1. <Space_1_+Space_2_+...+State>: This structure indicates concurrent symptoms in multiple anatomical locations (eg, 头/项/酸痛 [occipitocervical aching and headache]).
2. <Space+State_1_+State_2_+...>: This structure denotes the concurrent presence of 2 or more distinct symptoms within a single anatomical location, physiological product, or mental state (eg, 小/便/发黄/有泡沫 [dark-colored urine with foam formation]).
3. <Space_1_+State_1_+Space_2_+State_2_>: Within such structurally complex expressions, multiple Space entities coexist alongside multiple State entities. Comprehensive restoration of the symptoms requires a 2-by-2 combination of the Space and the State entities (eg, 头/晕/耳/鸣 [vertigo with concomitant tinnitus]).
4. <Space+Sev_Qual+State> and <Space+Freq+State>: In these 2 types of structurally complex expressions, between a specific anatomy (or physiological product) and a specific symptom, there are adjectives or adverbs to further describe the severity or frequency of the symptom in greater detail (eg, 头/有点/痛 [a mild headache], 大/便/经常/困难 [chronic defecatory difficulty]). To reconstruct the described symptom, other types of entities that exist between Space and State must be temporarily ignored, prioritizing the combination of the Space and State entities.
5. <Space+Neg+State_1_+State_2_+...>, <Neg+Symp_S_1_+Symp_S_2_+...>, <Neg+Symp_S+Space+State>, <Neg+Space_1_+State_1_+Space_2_+State_2_>: These complex structures are usually used to enumerate 2 or more negative symptoms (eg, 关节/无/红肿/热痛 [absence of joint erythema, swelling, hotness, or tenderness], 无/发热/恶寒 [absence of fever and chills]).

It is worth noting that the Space entity can be categorized into 2 distinct types: physical space and conceptual space. Physical space refers to entities that can be directly seen and touched, such as various parts of the human body and different physiological products, including urine, feces, sweat, and menstruation. Conceptual space, on the other hand, is an intangible, abstract existence that usually includes organ functions, numerous sensations, and mental activities. Thus, the Space entity that combines with the State entity can be further subdivided into the following five types: (1) an independent Pos_Pri (eg, 头 [head]); (2) Pos_SCP+Pos_Pri (eg, 小/便 [urine]); (3) an independent Pos_Sub (eg, 视物 [vision] and 皮肤 [skin]); (4) Pos_Pri+Pos_Sub (eg, 腕/关节 [wrist joint]); and (5) Pos_SCP+Pos_Pri+Pos_Sub (eg, 小/便/量 [urine volume]).

From the above combination structures, it can be seen that (1) the State entity cannot exist alone and must be concatenated with the previous Space entity to show clinical diagnostic value; (2) the Space entity that can be concatenated with the State entity is not limited to specific anatomy but also includes secretions such as urine and feces, as well as intangible physiological functions and mental states. The Space entity is broader than the Pos_Pri entity; (3) positive symptoms consist of 2 conceptual elements: Space and State, while negative symptoms are composed of 3 conceptual elements: Neg, Space, and State. Before outputting the concatenated results, it is necessary to check whether any negative symptoms have not yet been concatenated to avoid incorrectly converting them into positive ones.

**Figure 4.**
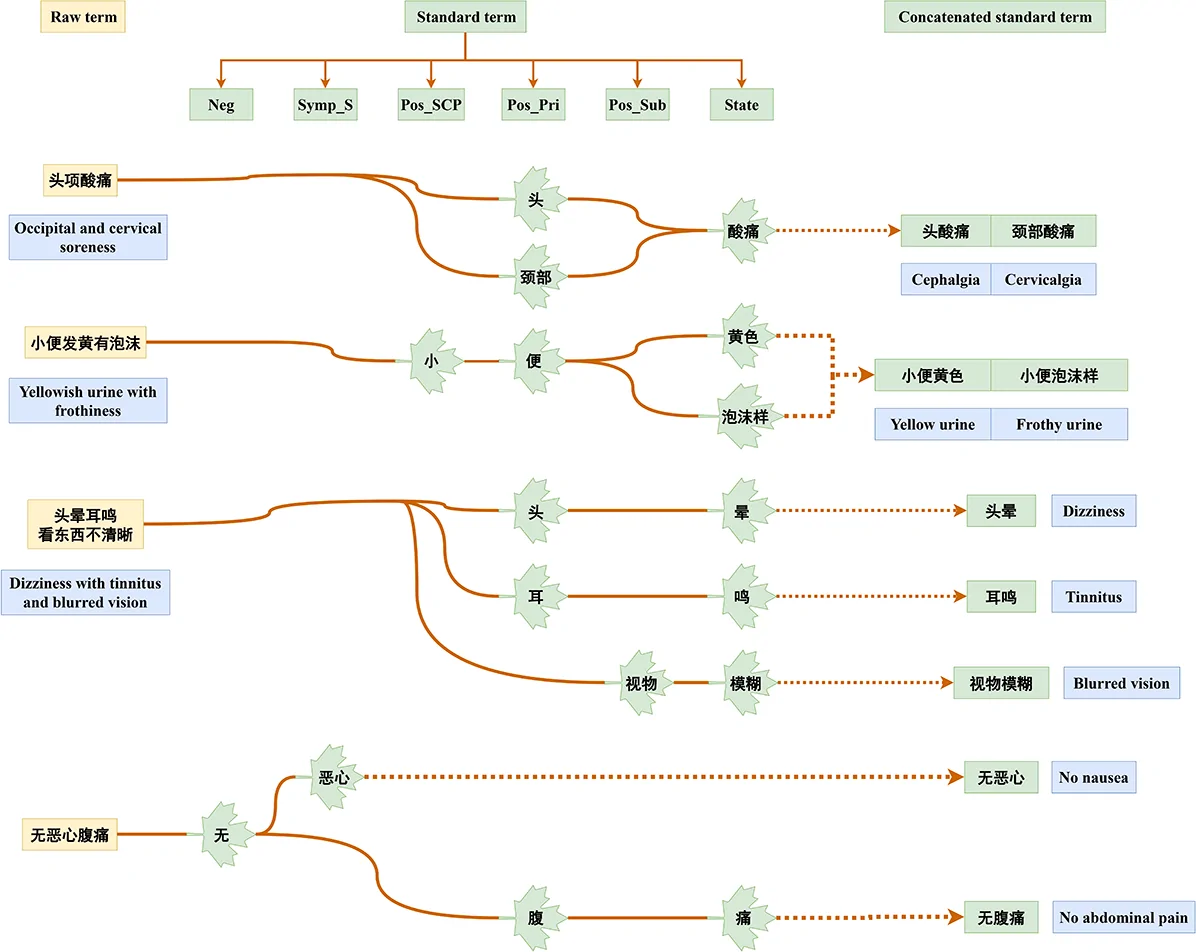
Trie tree concatenation method of standard terms.

Based on the above 3 core principles, we constructed a trie tree to concatenate the extracted standard terms: each branch (path) represents a complete symptom standard term. The leaves (start points of paths) are State or Symp_S entities. The roots (end points of paths) are Space or Neg entities, and each node on the branches (paths) has only one standard term. By collecting all branches (symptom standard terms), all symptoms in the complex expression can be extracted and comprehensively reconstructed. The trie tree concatenation method of standard terms is shown in Figure 4.

When the path starts from the State entity, it connects with the Pos_Pri or Pos_Sub node. If the node connected to the State entity is Pos_Sub, continue to determine whether it needs to connect with the Pos_Pri node, Pos_SCP node, and Neg node. If no additional nodes are available for concatenation, end the current path, and the symptom standard term becomes the output. When the node connected to the State node is Pos_Pri, continue to determine whether it needs to connect with the Pos_SCP node and Neg node.

**Figure 5.**
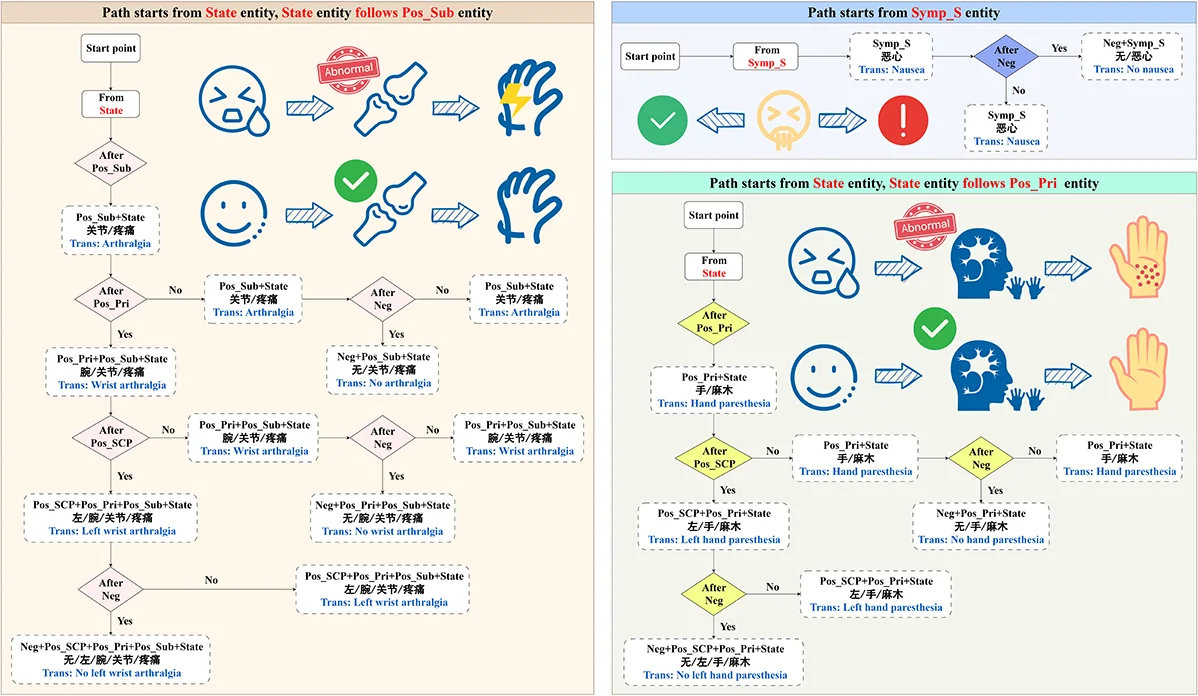
Flowchart of path generation.

When the path starts from a Symp_S entity and no Neg node can be used for concatenation in front of the Symp_S entity, the path can be ended directly, and the symptom standard term can be output. The specific method of path generation is shown in Figure 5.

### Sorting Rules of Standard Terms

Reordering standard terms yielded structured, coherent, and clinically meaningful outputs. Analysis of large-scale EMRs revealed that physicians typically follow a specific logical sequence when documenting: (1) onset time and precipitating factors; (2) symptom manifestations, severity, and frequency; and (3) disease progression. Therefore, after concatenating standard terms through the trie tree method, the final normalized output follows this order: Time entity, Cond entity, concatenated standard term, Sev_Qual entity, Sev_Quant entity, Freq entity, and Trend entity.

### Experimental Evaluation Indicators

The framework was evaluated at 2 stages, named entity recognition and named entity normalization, each with its own set of metrics. For the task of NER, model performance is commonly evaluated using precision, recall, and *F*_1_-score. The calculation of these metrics is based on the following classifications of the prediction results:

1. True positive (TP): represents the number of correctly identified entities.
2. True negative (TN): represents the number of correctly identified nonentities.
3. False positive (FP): represents the number of instances incorrectly identified as entities.
4. False negative (FN): represents the number of entities that the model failed to identify.

The specific formulas and their interpretations are shown in Table 3. As the *F*_1_-score provides a more comprehensive evaluation by considering both precision and recall, it was selected as the primary performance evaluation metric for the NER model in this study.

**Table 3. T3:** Formulas and interpretations of evaluation metrics for named entity recognition (NER).

Evaluation metric	Equation	Interpretation
Precision	$\text{P=}\frac{\text{TP}}{\text{TP+FP}}$	This metric, also known as positive predictive value, represents the proportion of true entities among all results identified as entities
Recall	$\text{R=}\frac{\text{TP}}{\text{TP+FN}}$	This metric, also known as sensitivity, indicates the proportion of all actual entities that are successfully identified by the model
*F*_1_-score	$F_{1}=\frac{\text{2PR}}{\text{P+R}}$	This metric is the harmonic mean of precision and recall

We then describe the metrics used for the normalization stage. For the NEN task, accuracy, precision, recall, and *F*_1_-score were used to evaluate model performance. Accuracy was calculated at the instance level using exact matching as the criterion. A prediction was considered correct only when the complete set of predicted standard terms is identical to the complete set of reference standard terms. Precision, recall, and *F*_1_-score were calculated at the standard-term level to further assess the ability of each model to retrieve individual standard terms. The formulas of these evaluation metrics are shown in Table 4.

**Table 4.** Formulas of evaluation metrics for named entity normalization (NEN).

Evaluation metric	Equation
Accuracy	$Acc=\frac{\text{Number of instances with completely correct prediction results}}{\text{Total number of test instances}}$
Precision	$P=\frac{\text{Number of correctly predicted standard terms}}{\text{Total number of standard terms output by the model}}$
Recall	$R=\frac{\text{Number of correctly predicted standard terms}}{\text{Total number of reference standard terms}}$
*F*_1_-score	$F_{1}=\frac{\text{2PR}}{\text{P+R}}$

To verify the performance differences between the proposed STC-NF model and the baseline models, the held-out test set generated from the 8:2 split of the 500 collected EMRs, as described in the “Introduction to Dataset Sources and Fine-Grained Semantic Classification” subsection, was further divided into 3 NEN test sets. First, 25 records were randomly selected, and the single-implication terms in these records were used to construct the single-implication test set. Second, another 25 records were randomly selected, and the multi-implication terms contained in these records were used to construct the multi-implication test set. Finally, the remaining 50 records were used to construct the mixed test set, which contained both single- and multi-implication terms. Therefore, the 3 test datasets were constructed from nonoverlapping EMRs, and the multi-implication terms in the mixed test set did not overlap with those in the multi-implication test set.

### Experimental Hyperparameters of the NEN Model

We used BERT-base-Chinese as the base model, which had a hidden layer dimensionality of 768. Because the NEN module formulated entity alignment as a sentence-pair semantic matching task between a raw term and a candidate standard term, the hyperparameter settings were determined by considering previous studies of medical concept normalization and biomedical entity normalization, as well as the short phrase-level characteristics of TCM symptom-term pairs. Recent Chinese medical concept normalization and rare disease concept normalization studies have shown that deep language models can effectively map nonstandard medical expressions to standardized medical concepts or vocabulary identifiers [45,46]. In a recent NER/NEN corpus study, transformer fine-tuning hyperparameters were selected on a development set, and a maximum sequence length of 128 was used for short biomedical entity mentions [47]. In addition, recent biomedical BERT fine-tuning experiments commonly evaluated learning rates of 1×10^–5^, 3×10^–5^, and 5×10^–5^, sequence lengths of 128, 256, and 512, batch sizes of 16 and 32, and dropout of 0.1 [48]. Therefore, we set the learning rate to 5×10^–5^, which falls within the commonly evaluated BERT fine-tuning range and allows efficient adaptation on a relatively small entity-alignment dataset. A dropout rate of 0.1 was used to reduce the risk of overfitting during supervised sentence-pair classification. The maximum sequence length was set to 128 because both the raw TCM symptom terms and candidate standard terms were short, phrase-level inputs; this setting was sufficient to cover the term pairs while reducing unnecessary computation. The training batch size was set to 30, close to the commonly used mini-batch range in biomedical BERT fine-tuning, while remaining compatible with the available GPU memory. The Adam optimizer was used for gradient-based parameter updates during BERT fine-tuning.

### Ethical Considerations

This study involving humans was approved by the Second Traditional Chinese Medicine Hospital of Guangdong Province, affiliated with the Guangzhou University of Chinese Medicine. The ethics approval number was Y202407-004-01. The study was conducted according to local legal and institutional requirements and adhered to the Declaration of Helsinki. This study was retrospective, and informed consent was waived.

## Results

### Named Entity Recognition Results

In this study, symptom terms were categorized into 12 different types of entities based on fine-grained semantic classification. Three NER models specialized for extracting symptom entities were trained according to customized annotation rules. The extraction performance of each model is shown in Table 5.

**Table 5.** Comparison of symptom entity extraction performance across models.

Entity type	BiLSTM^a^-CRF^b^ *F*_1_^c^-score ratio, (%)	BERT^d^-CRF *F*_1_-score ratio, (%)	BERT-BiLSTM-CRF *F*_1_-score ratio, (%)
Independent symptom (Symp_S)	1100/1186 (92.7)	1082/1186 (91.2)	1106/1192 (92.8)
Negative occurrence (Neg)	1112/1160 (95.9)	1088/1148 (94.8)	1102/1153 (95.6)
Scope space (Pos_SCP)	880/915 (96.2)	874/910 (96.0)	882/916 (96.3)
Primary space (Pos_Pri)	4324/4711 (91.8)	4316/4702 (91.8)	4340/4707 (92.2)
Secondary space (Pos_Sub)	348/370 (94.1)	344/365 (94.2)	348/368 (94.6)
Time of occurrence (Time)	816/888 (91.9)	796/880 (90.5)	804/884 (91.0)
Occurrence condition (Cond)	332/376 (88.3)	342/386 (88.6)	342/383 (89.3)
Frequency of occurrence (Freq)	416/446 (93.3)	406/441 (92.1)	414/443 (93.5)
Qualitative severity (Sev_Qual)	450/490 (91.8)	450/488 (92.2)	458/490 (93.5)
Quantitative severity (Sev_Quant)	90/103 (87.4)	92/105 (87.6)	92/104 (88.5)
Occurrence state (State)	4530/5124 (88.4)	4534/5112 (88.7)	4586/5141 (89.2)
Occurrence trend (Trend)	170/207 (82.1)	174/204 (85.3)	178/208 (85.6)

a BiLSTM: bidirectional long short-term memory.

b CRF: conditional random field.

c In every model column, each cell reports the *F*_1_-score as 2TP/(2TP+FP+FN) with the resulting percentage in parentheses (TP, FP, and FN [Table T3]as defined in the Experimental Evaluation Indicators subsection); this ratio is the *F*_1_-score calculation and does not represent the count of correctly recognized entities divided by the total number of entities.

d BERT: Bidirectional Encoder Representations from Transformers.

As shown in Table 5, the 3 models achieved comparable overall performance, although the best-performing model varied across entity types. BERT-BiLSTM-CRF obtained the highest or tied-highest *F*_1_-score for most entity types, achieving 4340 out of 4707 (92.2%) for Pos_Pri and 4586 out of 5141 (89.2%) for State among the high-frequency structural entities, as well as 1106 out of 1192 (92.8%) for Symp_S, 882 out of 916 (96.3%) for Pos_SCP, and 458 out of 490 (93.5%) for Sev_Qual. BiLSTM-CRF achieved the highest *F*_1_-scores for the Time and Neg entities, with *F*_1_-scores of 816 out of 888 (91.9%) and 1112 out of 1160 (95.9%), respectively, and remained within approximately one percentage point of the best-performing model on the high-frequency structural entities. The advantages of the BERT-based models over BiLSTM-CRF were concentrated on low-frequency modifier entities, such as Sev_Quant, Sev_Qual, and Trend, where the best-performing BERT-based model exceeded BiLSTM-CRF by 1.1, 1.7, and 3.5 percentage points, respectively. Complete per-entity precision, recall, and *F*_1_-scores for all three models are provided in Multimedia Appendix 3.

### Entity Alignment Results

We used 3 statistical models (TF-IDF, Med, and BM25), the multiclassification model PLM-*ICD*, the generative sequence-generation model, and the binary classification model MTCG as baseline models for comparison with our proposed STC-NF model. The experimental results of different normalization models on 3 test sets are shown in Table 6.

**Table 6.** Results of different normalization models on 3 test sets.

Model name and test set		Accuracy^a^, n/N (%)	Precision, n/N (%)	Recall, n/N (%)	*F*_1_-score, ratio (%)
TF-IDF^b^					
Single-implication		91/197 (46.2)	91/186 (48.9)	91/197 (46.2)	182/383 (47.5)
Multi-implication		0/369 (0.0)	83/328 (25.3)	83/1175 (7.1)	166/1503 (11.0)
Mixed		179/1124 (15.9)	344/1068 (32.2)	344/2225 (15.5)	688/3293 (20.9)
Med					
Single-implication		97/197 (49.2)	97/188 (51.6)	97/197 (49.2)	194/385 (50.4)
Multi-implication		0/369 (0.0)	88/331 (26.6)	88/1175 (7.5)	176/1506 (11.7)
Mixed		192/1124 (17.1)	366/1070 (34.2)	366/2225 (16.4)	732/3295 (22.2)
BM25					
Single-implication		106/197 (53.8)	106/191 (55.5)	106/197 (53.8)	212/388 (54.6)
Multi-implication		0/369 (0.0)	92/337 (27.3)	92/1175 (7.8)	184/1512 (12.2)
Mixed		210/1124 (18.7)	391/1082 (36.1)	391/2225 (17.6)	782/3307 (23.6)
PLM-*ICD*^c^					
Single-implication		168/197 (85.3)	168/184 (91.3)	168/197 (85.3)	336/381 (88.2)
Multi-implication		0/369 (0.0)	97/343 (28.3)	97/1175 (8.3)	194/1518 (12.8)
Mixed		326/1124 (29.0)	522/1102 (47.4)	522/2225 (23.5)	1044/3327 (31.4)
Sequence generation					
Single-implication		157/197 (79.7)	157/180 (87.2)	157/197 (79.7)	314/377 (83.3)
Multi-implication		189/369 (51.2)	593/1134 (52.3)	593/1175 (50.5)	1186/2309 (51.4)
Mixed		706/1124 (62.8)	1245/2142 (58.1)	1245/2225 (56.0)	2490/4367 (57.0)
MTCG^d^					
Single-implication		173/197 (87.8)	173/188 (92.0)	173/197 (87.8)	346/385 (89.9)
Multi-implication		223/369 (60.4)	698/1137 (61.4)	698/1175 (59.4)	1396/2312 (60.4)
Mixed		803/1124 (71.4)	1443/2156 (66.9)	1443/2225 (64.9)	2886/4381 (65.9)
STC-NF^e^					
Single-implication		180/197 (91.4)	180/192 (93.8)	180/197 (91.4)	360/389 (92.5)
Multi-implication		311/369 (84.3)	979/1141 (85.8)	979/1175 (83.3)	1958/2316 (84.5)
Mixed		990/1124 (88.1)	1931/2160 (89.4)	1931/2225 (86.8)	3862/4385 (88.1)

a Accuracy, precision, and recall are reported as n/N (%), whereas *F*_1_-score is reported as a ratio (%) calculated as 2TP/(2TP+FP+FN). Accuracy is calculated at the instance level using exact matching; precision, recall, and *F*_1_-score are calculated at the standard-term level. See Table 4 for the corresponding formulas.

b TF-IDF: term frequency-inverse document frequency.

c PLM-*ICD*: pretrained language model framework for *International Classification of Diseases* coding.

d MTCG: multi-task candidate generator.

e STC-NF: split-then-concatenate normalization framework.

As shown in Table 6, STC-NF achieved the best overall performance across the 3 test sets. On the single-implication test set, STC-NF achieved an accuracy of 180 out of 197 (91.4%), a precision of 180 out of 192 (93.8%), a recall of 180 out of 197 (91.4%), and an *F*_1_-score of 360 out of 389 (92.5%). Compared with PLM-*ICD* and MTCG, which were the stronger baseline models for single-implication terms, STC-NF improved accuracy by 6.1 and 3.6 percentage points, respectively.

For the multi-implication test set, the accuracy of TF-IDF, Med, BM25, and PLM-*ICD* was 0 out of 369 (0.0%). This result was attributable to the single-output design of these models, which prevented them from producing a complete set of standard terms for multi-implication symptom terms. However, their term-level precision, recall, and *F*_1_-score were still calculated to reflect partial standard-term retrieval performance. In contrast, STC-NF achieved an accuracy of 311 out of 369 (84.3%), a precision of 979 out of 1141 (85.8%), a recall of 979 out of 1175 (83.3%), and an *F*_1_-score of 1958 out of 2316 (84.5%). Compared with sequence generation and MTCG, STC-NF improved multi-implication accuracy by 33.1 and 23.9 percentage points, respectively.

On the mixed test set, STC-NF achieved an accuracy of 990 out of 1124 (88.1%), a precision of 1931 out of 2160 (89.4%), a recall of 1931 out of 2225 (86.8%), and an *F*_1_-score of 3862 out of 4385 (88.1%). These results were higher than those of all baseline models. Compared with MTCG, the best-performing baseline model on the mixed test set, STC-NF improved accuracy by 16.7 percentage points and *F*_1_-score by 22.2 percentage points. These findings indicate that the split-then-concatenate mechanism improves not only complete instance-level normalization accuracy but also standard-term-level retrieval performance, particularly for complex multi-implication symptom terms.

### Structured Normalization Results

After identifying the standard terms with the same concept as each raw term through a BERT-based binary classification model, this study concatenated and reordered standard terms with specific semantic labels according to predefined rules and output structured text to complete the normalization of TCM symptom terminology. To improve the readability of the main text and avoid an overly crowded bilingual table, Tables 7 and 8 present the English versions of the structured normalization results. The corresponding original Chinese raw terms, segmentation results, and structured normalization outputs are available in Multimedia Appendix 4.

**Table 7.** Translated examples of raw symptom expressions and their normalized symptom terms.

Number	Raw term	Concatenated standard term
1	Occipitocervical aching and headache have been reported in recent days, without significant improvement following rest.	Headache and neck pain
2	Mild exertional chest tightness and dyspnea on stair climbing have persisted for several weeks.	Chest tightness and shortness of breath
3	An unintentional weight loss of approximately 5 kg has occurred over the past 6 months.	Weight loss
4	Severe persistent odynophagia has been noted without associated chills or fever.	Sore throat without chills or fever

**Table 8.** Structured semantic attributes corresponding to the examples in Table 7.

Number	Time	Cond	Sev_Qual	Sev_Quant	Freq	Trend
1	Acute symptom	—^a^	—	—	—	Not improving
2	Subacute symptom	Exertion-induced	Mild	—	—	—
3	Chronic symptom	—	—	Approximately 5 kg	—	—
4	—	—	Severe	—	Persistent symptom	—

a Not applicable.

## Discussion

### Performance Comparison of Named Entity Recognition Models

In the experiments of recognizing symptom entities, BiLSTM-CRF achieved the highest *F*_1_-scores among the 3 models for the Time and Neg entities, at 91.9% and 95.9%, respectively. For several entity types, including Pos_Pri, Pos_Sub, and State, the differences in *F*_1_-scores among BiLSTM-CRF, BERT-CRF, and BERT-BiLSTM-CRF were small (within about one percentage point), with BiLSTM-CRF reaching an *F*_1_-score of 91.8% for the Pos_Pri entity. Importantly, although the BERT-based models obtained higher *F*_1_-scores for most entity types, their largest gains over BiLSTM-CRF were concentrated on low-frequency modifier entities, such as Sev_Quant, Sev_Qual, and Trend. In contrast, for the high-frequency structural entities that dominate downstream symptom reconstruction—most notably Pos_Pri and State—BiLSTM-CRF was essentially on par with the pretrained models, trailing by only about 0.4 and 0.8 percentage points, respectively. This narrow gap can be attributed to the fine-grained semantic classification scheme adopted in this study. By constraining each category to short, structurally regular spans with clearly delimited boundaries, the annotation rules reduce intracategory description diversity and lower the decision dimensionality of the recognition model so that a lightweight sequence model is already able to capture the local dependencies required for accurate boundary and type prediction. Consequently, the marginal benefit obtainable from large-scale pretraining is limited under the present annotation scheme. Xue et al [49] and Li et al [50] proposed the porous lattice transformer encoder (PLTE) and flat-lattice transformer (FLAT), respectively, for Chinese NER, showing that incorporating external lexicon information through character-word lattices can effectively exploit word-boundary cues and improve Chinese NER performance. These studies suggest that lexicon-enhanced transformer models are valuable when word-boundary ambiguity and external word information are major factors affecting entity recognition. However, the objective of the NER stage in STC-NF was not to pursue the highest possible benchmark score but to provide stable fine-grained semantic units for subsequent label-constrained entity alignment and rule-guided reconstruction. Considering the recognition performance reported above, its lower model complexity (specifically, substantially fewer parameters and no dependence on large-scale pretrained weights), and the downstream requirement for stable entity decomposition, BiLSTM-CRF was selected as the NER component of STC-NF. Although the BERT-based models achieved marginally higher *F*_1_-scores on most entity types in Table 5, the largest gains occurred on low-frequency modifier entities, which are peripheral to symptom reconstruction and can be further rectified by the downstream concatenation and sorting rules. Therefore, this advantage does not warrant their substantially higher computational cost in the present 2-stage pipeline. More advanced lexicon-enhanced transformer architectures, such as PLTE and FLAT, remain promising alternatives for future evaluation on larger and more heterogeneous TCM clinical corpora.

### Merits and Limitations of Baseline Models

Although statistical models have the advantage of fast processing, they are not competitive with deep learning models in semantic understanding. Clinical term normalization studies have shown that nonstandard clinical expressions often differ from standardized terminology because of synonyms, stylistic variation, morphological variation, abbreviations, and local writing conventions, making surface-form matching insufficient for robust normalization [51]. Similarly, recent biomedical NER and NEN systems have combined neural NER with neural or hybrid normalization modules to overcome the inability of dictionary- or rule-only methods to cover complex mention variations [52]. Therefore, the relatively weak performance of TF-IDF, Med, and BM25 in this study was mainly attributable to their dependence on character-level or lexical similarity rather than contextual semantic matching.

The multiclassification model PLM-*ICD* first segments the raw text into sentences. After embedding each sentence and all *ICD* codes into dense vectors, it selects the *ICD* embedding with the highest cosine similarity (computed using attention-weighted BERT or BioBERT models pretrained on corpora, such as Medical Information Mart for Intensive Care III [MIMIC-III]), to align the raw term. PLM-*ICD* performed well for single-implication terms. However, because of its multiclassification design, the model could output only one prediction for each input sentence, regardless of how many symptoms were included. Therefore, PLM-*ICD* could not handle multi-implication terms such as “腰酸背痛 (lumbago and back pain with soreness),” “尿频量多 (polyuria with increased urinary frequency),” and so on.

Generative model sequence generation first uses a character-level text generation model built upon transformer to convert each character in the raw term step by step. Once multiple candidate entity sets (sentences) of standard terms are generated, a pretrained BERT model calculates the semantic similarity between the input text and each set. Then, the top-n generated sets are chosen as the final normalization results after reordering. Compared to PLM-*ICD*, this model demonstrates the capability to handle multi-implication terms. However, the single-implication accuracy is significantly lower, as splitting the raw term word by word sometimes destroys the structural integrity of the entity. For example, if “恶心 (nausea)” is split into “恶 (hate)” and “心 (heart)” and then converted and concatenated together, the final result will be the wrong prediction of “厌恶心脏 (loathe heart).”

The binary classification model MTCG significantly reduces the number of semantic comparisons through its retrieval strategy, resulting in higher entity alignment efficiency than the multiclassification approach. In addition, it achieved higher accuracy in dealing with single-implication terms such as “恶心 (nausea),” “发热 (fever),” “咳 (cough),” etc, which contain only one symptom, because it does not split the raw term and retains the structural integrity of the entity, compared to the sequence-generation approach. A key limitation of this model is its misclassification of compound symptoms that have unclear entity boundaries. The advantage of STC-NF over MTCG was most evident for multi-implication terms. Unlike MTCG, STC-NF explicitly decomposes multi-implication expressions before semantic matching and reconstructs the normalized outputs after alignment. This additional split-and-recombine process helps preserve multiple symptom concepts within a single raw phrase, which cannot always be represented by a single retrieved standard term. Taking “尿频量多 (polyuria with increased urinary frequency)” as an example, since “量多 (high volume)” does not immediately follow “尿 (urine),” MTCG recognized only the raw term “尿频 (frequent urination)” but omitted another raw term “尿量多 (excessive urination)” at the end. Therefore, although MTCG exhibits remarkable generalization performance and excellent accuracy on the mixed test set, a significant gap remains between MTCG and our model when handling multi-implication terms.

### Mechanistic Explanation for the Superior Performance of STC-NF

Overall, the superior performance of STC-NF can be explained by the complementarity of its 3 components. First, fine-grained semantic extraction decomposes complex symptom expressions into clinically meaningful semantic units, thereby reducing the boundary ambiguity caused by overlapping, discontinuous, and nested entities. This is consistent with recent medical NER evidence showing that entity phrase length and the number of words in an entity phrase can substantially influence recognition performance [53], and with systematic evidence that discontinuous clinical entities remain challenging for traditional NER methods [54]. Second, the BERT-based binary classification module preserves semantic matching at the concept-alignment stage instead of relying only on surface similarity. Third, the trie tree–based concatenation rules explicitly reconstruct multi-implication symptom terms after semantic alignment. This design is consistent with 2-stage clinical text workflows that first extract information from free-text notes and then harmonize extracted mentions into standardized database concepts [55], as well as recent hybrid neural-symbolic clinical NLP systems that integrate neural recognition with structured vocabularies and symbolic reasoning to transform unstructured clinical notes into standardized terms [56]. Therefore, STC-NF improved not only complete instance-level normalization accuracy but also standard-term-level retrieval performance, especially for multi-implication symptom terms with complex internal structures.

### Limitations and Future Work

This study has several limitations that suggest avenues for future research. First, the NEN module used BERT-base-Chinese as the base semantic-matching model, without evaluating more recent clinical or biomedical language–modeling strategies. Indirect evidence from our previous work on the upstream NER stage suggests that the choice of pretrained backbone is important for fine-grained TCM symptom modeling. In that study, we evaluated several external NER backbone models on fine-grained TCM symptom corpora, including BERT-base-Chinese+CRF, BERT-base-Chinese+BiLSTM+CRF, Chinese-RoBERTa-wwm-ext+CRF, and Chinese-RoBERTa-wwm-ext+BiLSTM+CRF. The results showed that Chinese-RoBERTa-wwm-ext+BiLSTM+CRF achieved higher NER *F*_1_-scores than BiLSTM+CRF on both GDTCM-500 and HwaMei-500, suggesting that more advanced pretrained language models may further improve symptom entity recognition [57]. However, these experiments were limited to the entity recognition stage. We have not yet extended these external backbone comparisons to the full NEN stage, because such validation would require reconstructing entity-alignment samples, rerunning the complete split-then-concatenate pipeline, and performing additional expert validation of normalized outputs. Therefore, future work will compare STC-NF with more advanced pretrained language models, retrieval-augmented models, and domain-specific clinical language models at both the NER and NEN stages to determine whether the proposed split-then-concatenate mechanism remains beneficial across different backbone architectures. Accordingly, these results should be interpreted as evidence for the effectiveness of the split-then-concatenate mechanism under a BERT-base-Chinese alignment setting, rather than as evidence that BERT-base-Chinese is the optimal backbone for the NEN module.

Second, the generalizability of the model may be constrained, as the training data were sourced entirely from a single clinical department (endocrinology). The framework’s effectiveness has not yet been validated using multisource data from departments such as surgery or pediatrics. This limitation is particularly important because model robustness depends not only on algorithmic structure but also on how heterogeneous information sources are represented and transferred across contexts. Beyond medical applications, the methodological idea of quantifying free-text content and integrating it with structured variables in decision-support pipelines has been demonstrated in other domains as well, for example, by Wang et al [58] in a consumer-review setting. More directly within health care, Hou et al [59] highlighted in a chronic disease prediction setting that multimodal electronic health record representation learning and domain adaptation can improve predictive generalizability under cross-domain distribution shifts. These studies suggest that future versions of STC-NF should not only expand the corpus to multiple clinical departments but also explore whether domain adaptation and multimodal representation learning can improve the robustness of TCM symptom terminology normalization across heterogeneous EMR sources.

Finally, the construction of positive samples and standard knowledge bases still relied partly on manual expert curation, especially for infrequent entities. This process ensured semantic accuracy but limited scalability. Recent work on medical concept annotation has noted that supervised medical concept normalization often requires sufficient annotated data, whereas the availability of annotated clinical data is constrained by privacy, sensitivity, and the time required for expert annotation [60]. Similarly, clinical registry-oriented NLP reviews have identified manual extraction and annotation as labor- and resource-intensive steps that may hinder large-scale deployment [61]. To reduce this bottleneck, future work will explore semiautomatic candidate generation and weakly supervised concept annotation. In addition, the distinctive features of Chinese radicals may provide useful linguistic cues for TCM symptom terminology. For example, Chinese characters containing the radical “疒” are often related to State entities, such as “疼 (painful),” “瘫 (paralytic),” and “疯 (mad),” whereas characters containing the radical “月” are often found in Pos_Pri entities related to anatomy, such as “胸 (chest),” “腹 (abdomen),” and “肢 (limb).” Incorporating radical-aware representations into language models may reduce the candidate screening range and improve the efficiency of positive sample construction.

### Conclusions

This study proposed the STC-NF, a 2-stage deep learning approach that uses fine-grained semantic classification to normalize TCM symptom terminology. The STC-NF model achieved an accuracy of 180 out of 197 (91.4%) on the single-implication test set, 311 out of 369 (84.3%) on the multi-implication test set, and 990 out of 1124 (88.1%) on the mixed test set. In addition, the corresponding *F*_1_-scores reached 360 out of 389 (92.5%), 1958 out of 2316 (84.5%), and 3862 out of 4385 (88.1%), respectively. These results demonstrate that the split-then-concatenate mechanism substantially improves both instance-level normalization accuracy and standard-term-level retrieval performance for TCM symptom terminology normalization.

Beyond its quantitative performance, the proposed framework facilitates the automatic extraction and structuring of key clinical information from unstructured EMRs, thereby enabling the development of advanced applications. In clinical practice, these structured data can enable intelligent analyses to support clinicians in diagnosis and treatment. By converting diverse and ambiguous patient-reported symptoms into standardized terms, the framework provides a consistent data foundation for clinical decision support systems. This uniformity allows for large-scale analysis to identify symptom patterns predictive of specific conditions, potentially reducing diagnostic ambiguity and enhancing the precision of care.

Furthermore, this data structuring capability is instrumental for constructing high-quality biomedical knowledge bases and knowledge graphs. The normalization process transforms raw textual descriptions into discrete, standardized entities, a prerequisite for building robust and interoperable knowledge representations. Such knowledge graphs can map complex relationships among symptoms, diseases, and treatments, potentially facilitating the development of sophisticated tools for intelligent health care and personalized medicine. However, practical deployment of this framework still requires sufficiently representative annotated EMR data, continuously updated standard knowledge bases, and further validation across heterogeneous clinical departments.

Finally, this work has significant implications for health care administration, particularly for medical insurance cost control and diagnosis-related group (DRG) assignment. Hospital reimbursement through DRGs depends in part on accurate diagnostic coding derived from clinical evidence, including patient symptoms. The heterogeneity in raw symptom descriptions can lead to inconsistent coding and inaccurate DRG assignments. By standardizing symptom terminology, our framework produces uniform, machine-readable data that enhance diagnostic reliability. This improvement fosters more consistent and accurate coding, which is fundamental to a precise DRG system. Consequently, our approach supports a more scientific and equitable assessment of hospital management and service quality, ensuring that reimbursement accurately reflects patient case complexity and contributing to the advancement of medical informatization.

## Supplementary material

10.2196/85825 Multimedia Appendix 1 Examples of the positive sample dataset.

10.2196/85825 Multimedia Appendix 2 Introduction to positive sample data augmentation methods.

10.2196/85825 Multimedia Appendix 3 Complete per-entity precision, recall, and *F*_1_-score values for all models.

10.2196/85825 Multimedia Appendix 4 Original Chinese examples of structured normalization results.

## Abbreviations

AutoML: automated machine learning
BERT: Bidirectional Encoder Representations from Transformers
BiLSTM: bidirectional long short-term memory
CFFM: correlated feature fusion module
CRF: conditional random field
DRG: diagnosis-related group
DSG: directional skip-gram
EMR: electronic medical record
ERNIE: enhanced representation through knowledge integration
FLAT: flat-lattice transformer
FN: false negative
FP: false positive
HLT: hierarchical labeling tree
*ICD-10*: *International Statistical Classification of Diseases, 10th Revision*
MIMIC-III: Medical Information Mart for Intensive Care III
MTCG: multi-task candidate generator
NEN: named entity normalization
NER: named entity recognition
NLP: natural language processing
OCMH: output comparison of multiple heterogeneous models
PLM-*ICD*: pretrained language model framework for *International Classification of Diseases* coding
PLTE: porous lattice transformer encoder
SNOMED CT: Systematized Nomenclature of Medicine Clinical Terms
STC-NF: split-then-concatenate normalization framework
STC-TC: synonymous term conversion with thinking of candidate terms
symNormHS: symptom terminology normalization approach with hierarchical semantics
TCM: traditional Chinese medicine
TF-IDF: term frequency-inverse document frequency
TN: true negative
TP: true positive
WHO: World Health Organization
