# Supplementary material for: Symptom Terminology Normalization in Traditional Chinese Medicine: Development and Evaluation of a 2-Stage Deep Learning Framework Based on Fine-Grained Semantic Classification
Source: JMIR Med Inform. 2026 Sep 25;14:e85825. doi: 10.2196/85825 (PMC13614512; doi:10.2196/85825)
Supplement: Supplementary file 2 [file medinform-v14-e85825-s002.doc]

**Examples of the Positive Sample Dataset**

The core of symptom terminology normalization lies in constructing positive samples for training the binary classification model. Specific examples of the positive sample dataset are provided in Table 1.

**Table 1.** Specific examples of the positive sample dataset.

| **Raw Term** | **Standard Term** |
| --- | --- |
| 肺痨(phthisis) | 肺结核(tuberculosis) |
| 花柳病(venereal disease) | 梅毒(syphilis) |
| 消渴(diabetes) | 糖尿病(diabetes mellitus) |
| 老年痴呆(senile dementia) | 阿尔茨海默病(Alzheimer's disease) |
| 心窝(solar plexus) | 上腹部剑突下  (upper abdomen, below the xiphoid process) |
| 青筋(blue vein) | 浅表静脉(superficial vein) |
| 24-hour urine output ≥ 3000 mL | 多尿(polyuria) |
| Hourly urine output ≤ 17 mL | 少尿(oliguria) |
| 24-hour urine output ≤ 100 mL | 无尿(anuria) |
| Bowel movements more than  3 times per day | 便秘(constipation) |
| 37°C | 体温正常(normal body temperature) |
| 40℃ | 高热(high fever) |
| 针扎样痛(pain like a needle prick) | 刺痛(stabbing pain) |
| 痛而喜暖(pain relieved by warmth) | 冷痛(pain with a cold sensation) |
| 两边(right and left) | 双侧(bilateral) |
| 看东西(vision) | 视力(visual acuity) |
| 近半年来(over the past six months) | 慢性症状(chronic symptom) |
| 昨晚(last night) | 急性症状(acute symptom) |
| 1月余(over a month) | 亚急性症状(subacute symptom) |
| 上楼梯后出现(after climbing stairs) | 劳累后发作(exertion-induced) |
| 空腹时(when fasting) | 饥饿时发作(when hungry) |
| 经常出现(frequently occurring) | 复发性症状(recurrent symptom) |
| 时不时(occasionally) | 间歇性症状(intermittent symptom) |
| 一直(always) | 持续性症状(persistent symptom) |
| 每晚2-3次(2-3 times per night) | 频率增加(increased frequency) |
| 三日一行(once every three days) | 频率减少(decreased frequency) |
| 未见明显改善  (no significant improvement) | 无好转(no improvement) |
| 次数明显增多  (significantly increased frequency) | 恶化(worsening) |
| 得到控制(under control) | 好转(improvement) |
| 症状消失(symptom disappears) | 痊愈(recovery) |
