# Supplementary material for: Symptom Terminology Normalization in Traditional Chinese Medicine: Development and Evaluation of a 2-Stage Deep Learning Framework Based on Fine-Grained Semantic Classification
Source: JMIR Med Inform. 2026 Sep 25;14:e85825. doi: 10.2196/85825 (PMC13614512; doi:10.2196/85825)
Supplement: Supplementary file 3 [file medinform-v14-e85825-s003.doc]

**Introduction to Positive Sample Data Augmentation Methods**

Negative samples can be generated in bulk using exclusion methods and specific filtering rules without manual pairing. To address the issue of imbalanced distribution between positive and negative samples, this study proposed five data augmentation methods. Specific operational examples are provided in Table 1.

**Table 1.** Specific data augmentation methods for positive samples.

| **Method** | **Description** | **Example** |
| --- | --- | --- |
| Replace | Randomly replace Chinese characters in raw terms with numbers, and numbers with Chinese characters. | 1日3次→一日三次  (3 times a day) |
|  | Using the Synonyms Chinese Synonym Toolkit, replace adverbs or adjectives in raw terms with their synonyms. | 无法站立→不能站立(unable to stand) |
| Merge | Merge two entities sharing the same classification label and identical characters, then remove duplicate characters after merging. | 脑袋+脑瓜→脑袋瓜  ("脑袋" and "脑瓜" are Pos_Pri entities,  both meaning "head".) |
| Insert | Attaching meaningless suffixes like "儿" or  "子" to the end of an entity. | 后脑勺→后脑勺儿  (back of the head) |
|  | Inserting meaningless structural particles like  "的" or "得" that carry no substantive meaning into the middle of an entity. | 针刺样痛→针刺样的痛  (pain like a needle prick) |
| Combine | After "Merge", use the "Insert" method. | ①脑袋+脑瓜→"Merge"  →脑袋瓜;②脑袋瓜→  "Insert"→脑袋瓜子 |
| Copy | Copy Chinese characters from the entity to form an "AABB" pattern reduplicated word. | 反复→反反复复  (repeatedly) |
