# Supplementary material for: Symptom Terminology Normalization in Traditional Chinese Medicine: Development and Evaluation of a 2-Stage Deep Learning Framework Based on Fine-Grained Semantic Classification
Source: JMIR Med Inform. 2026 Sep 25;14:e85825. doi: 10.2196/85825 (PMC13614512; doi:10.2196/85825)
Supplement: Supplementary file 4 [file medinform-v14-e85825-s004.doc]

**Complete Per-Entity Precision, Recall, and F1 Values for All Models**

Table 5 in the main text reports the F1 score achieved by each model for every entity type. This appendix provides the complete precision (P), recall (R), and F1 score results of the three models—BiLSTM-CRF, BERT-CRF, and BERT-BiLSTM-CRF—for each of the 12 symptom entity types, as shown in Table 1.

**Table 1.** Complete precision, recall, and F1 values of the three models for each entity type.

| **Entity Type** | **Model** | **P** | **R** | **F1** **score** |
| --- | --- | --- | --- | --- |
| Symp_S | BiLSTM-CRF | 550/589 (93.4%) | 550/597 (92.1%) | 1100/1186 (92.7%) |
| Symp_S | BERT-CRF | 541/589 (91.9%) | 541/597 (90.6%) | 1082/1186 (91.2%) |
| Symp_S | BERT-BiLSTM-CRF | 553/595 (92.9%) | 553/597 (92.6%) | 1106/1192 (92.8%) |
| Neg | BiLSTM-CRF | 556/579 (96.0%) | 556/581 (95.7%) | 1112/1160 (95.9%) |
| Neg | BERT-CRF | 544/567 (95.9%) | 544/581 (93.6%) | 1088/1148 (94.8%) |
| Neg | BERT-BiLSTM-CRF | 551/572 (96.3%) | 551/581 (94.8%) | 1102/1153 (95.6%) |
| Pos_SCP | BiLSTM-CRF | 440/459 (95.9%) | 440/456 (96.5%) | 880/915 (96.2%) |
| Pos_SCP | BERT-CRF | 437/454 (96.3%) | 437/456 (95.8%) | 874/910 (96.0%) |
| Pos_SCP | BERT-BiLSTM-CRF | 441/460 (95.9%) | 441/456 (96.7%) | 882/916 (96.3%) |
| Pos_Pri | BiLSTM-CRF | 2162/2355 (91.8%) | 2162/2356 (91.8%) | 4324/4711 (91.8%) |
| Pos_Pri | BERT-CRF | 2158/2346 (92.0%) | 2158/2356 (91.6%) | 4316/4702 (91.8%) |
| Pos_Pri | BERT-BiLSTM-CRF | 2170/2351 (92.3%) | 2170/2356 (92.1%) | 4340/4707 (92.2%) |
| Pos_Sub | BiLSTM-CRF | 174/185 (94.1%) | 174/185 (94.1%) | 348/370 (94.1%) |
| Pos_Sub | BERT-CRF | 172/180 (95.6%) | 172/185 (93.0%) | 344/365 (94.2%) |
| Pos_Sub | BERT-BiLSTM-CRF | 174/183 (95.1%) | 174/185 (94.1%) | 348/368 (94.6%) |
| Time | BiLSTM-CRF | 408/458 (89.1%) | 408/430 (94.9%) | 816/888 (91.9%) |
| Time | BERT-CRF | 398/450 (88.4%) | 398/430 (92.6%) | 796/880 (90.5%) |
| Time | BERT-BiLSTM-CRF | 402/454 (88.5%) | 402/430 (93.5%) | 804/884 (91.0%) |
| Cond | BiLSTM-CRF | 166/176 (94.3%) | 166/200 (83.0%) | 332/376 (88.3%) |
| Cond | BERT-CRF | 171/186 (91.9%) | 171/200 (85.5%) | 342/386 (88.6%) |
| Cond | BERT-BiLSTM-CRF | 171/183 (93.4%) | 171/200 (85.5%) | 342/383 (89.3%) |
| Freq | BiLSTM-CRF | 208/221 (94.1%) | 208/225 (92.4%) | 416/446 (93.3%) |
| Freq | BERT-CRF | 203/216 (94.0%) | 203/225 (90.2%) | 406/441 (92.1%) |
| Freq | BERT-BiLSTM-CRF | 207/218 (95.0%) | 207/225 (92.0%) | 414/443 (93.5%) |
| Sev_Qual | BiLSTM-CRF | 225/244 (92.2%) | 225/246 (91.5%) | 450/490 (91.8%) |
| Sev_Qual | BERT-CRF | 225/242 (93.0%) | 225/246 (91.5%) | 450/488 (92.2%) |
| Sev_Qual | BERT-BiLSTM-CRF | 229/244 (93.9%) | 229/246 (93.1%) | 458/490 (93.5%) |
| Sev_Quant | BiLSTM-CRF | 45/52 (87%) | 45/51 (88%) | 90/103 (87.4%) |
| Sev_Quant | BERT-CRF | 46/54 (85%) | 46/51 (90%) | 92/105 (87.6%) |
| Sev_Quant | BERT-BiLSTM-CRF | 46/53 (87%) | 46/51 (90%) | 92/104 (88.5%) |
| State | BiLSTM-CRF | 2265/2565 (88.3%) | 2265/2559 (88.5%) | 4530/5124 (88.4%) |
| State | BERT-CRF | 2267/2553 (88.8%) | 2267/2559 (88.6%) | 4534/5112 (88.7%) |
| State | BERT-BiLSTM-CRF | 2293/2582 (88.8%) | 2293/2559 (89.6%) | 4586/5141 (89.2%) |
| Trend | BiLSTM-CRF | 85/97 (88%) | 85/110 (77.3%) | 170/207 (82.1%) |
| Trend | BERT-CRF | 87/94 (93%) | 87/110 (79.1%) | 174/204 (85.3%) |
| Trend | BERT-BiLSTM-CRF | 89/98 (91%) | 89/110 (80.9%) | 178/208 (85.6%) |
